# Process Development of Fly Ash-Based Geopolymer Mortars in View of the Mechanical Characteristics

**DOI:** 10.3390/ma14112935

**Published:** 2021-05-29

**Authors:** Hatice Öznur Öz, Neslihan Doğan-Sağlamtimur, Ahmet Bilgil, Aykut Tamer, Kadir Günaydin

**Affiliations:** 1 Department of Civil Engineering, Niğde Ömer Halisdemir University, Niğde 51240, Turkey; abilgil@ohu.edu.tr; 2 Department of Environmental Engineering, Niğde Ömer Halisdemir University, Niğde 51240, Turkey; neslihandogansaglamtimur@gmail.com; 3 Department of Mechanical Engineering, Imperial College London, London SW7 2AZ, UK; a.tamer@imperial.ac.uk; 4 Department of Aerospace Science and Technology, Politecnico di Milano, 20156 Milano, Italy; kadir.gunaydin@polimi.it

**Keywords:** environmentally friendly sustainable material, numerical analysis, fly ash, geopolymer, SEM-EDX, TGA-DTA, XRD, reuse, waste

## Abstract

This study aimed to determine the effects of design parameters, including the liquid/solid ratio (L/S), Na_2_SiO_3_/NaOH weight ratio, and curing temperature, on class F fly ash-based geopolymer composites. For this purpose, two disparate sources of fly ash were supplied from Çatalağzı (FA) and İsken Sugözü (FB) Thermal Power Plants in Turkey. Two different L/S ratios of 0.2 and 0.4 were used. The Na_2_SiO_3_/NaOH ratios in the alkaline solutions were 1, 1.5, 2, 2.5, and 3 by weight for each type of geopolymer mixture. Then, 40 different mixes were cured at two specific temperatures (70 °C and 100 °C) for 24 h and then preserved at room temperature until testing. Thereafter, the physical water absorption properties, apparent porosity, and bulk density were examined at 28 days on the hardened mortars. Additionally, compressive and flexural tests were applied to the geopolymers at 7, 28, and 90 days. It was found that the highest compressive strength was 60.1 MPa for the geopolymer manufactured with an L/S of 0.2 and Na_2_SiO_3_/NaOH ratio of 2. Moreover, the best thermal curing temperature for obtaining optimal strength characteristics was 100 °C for the FB.

## 1. Introduction

Ordinary Portland cement (OPC) is the essential material and compound in the construction industry due to the strength requirements for structural concrete. The increased production of OPC to fulfill the extensive demand for concrete has resulted in high energy consumption and depletion of natural sources, deteriorating the ecological balance. Additionally, cement production has become a leading cause of greenhouse gas emissions [1]. Approximately 4 billion tons of CO_2_ are emitted annually worldwide during the production of cement, which accounts for 7% of the overall CO_2_ emissions [1,2,3]. Therefore, new types of binders must be developed as a sustainable replacement to OPC to minimize environmental pollution in the construction business [4,5]. 

Industrial processes produce several million tons of solid waste each year. Most of the resulting waste falls into disuse and causes storage problems. Utilization of industrial wastes, such as fly ash (F), blast furnace slag, and silica fume, as cement replacements is advantageous for reducing the demand for large quantities of cement, whose production is the main factor in the emission of greenhouse gases [6,7]. In addition to limiting the use of cement, increasing interest in green concrete has driven researchers to investigate sustainable construction materials. A candidate material is geopolymer, which was initially presented in 1979 by Davidovits [8]. Geopolymer is a composition of large amounts of silica and alumina as the base materials, which is activated by adopting alkaline liquid as a binder [9,10]. The process is referred to as geopolymerization. During the geopolymerization process, a strongly alkaline medium comprising a simple or combined alkaline solution, i.e., an alkaline activator, is necessary to dissolve the silica in a certain amount of alumina and to enhance the surface hydrolysis of particles for raw materials [11]. Sodium hydroxides (NaOH), sodium silicate (Na_2_SiO_3_), potassium hydroxide (KOH), and potassium silicate are the most prevalent materials utilized as activators to synthesize geopolymers. Geopolymers possess valuable mechanical and corrosive properties, such as good compressive strength, fine durability against sodium and magnesium sulfate solutions, minimal shrinkage, and elevated resistance to acid attacks [8,12,13]. Furthermore, high temperature (above 100 °C) is not necessary for calcination during the production of the geopolymer. Finally, geopolymers can be obtained from industrial by-products with low energy consumption. As a result, the amount of greenhouse gas released to the environment is 44–64% less than that emitted during the production of OPC [14,15]. Therefore, geopolymers are considered environmentally friendly materials.

Geopolymerization consists of four steps: (1) dissolution in an alkaline activator; (2) formation of small coagulated structures; (3) polycondensation of soluble species; and (4) bonding of undissolved solid particles [16,17]. The properties of geopolymers are mainly dictated by the properties of the base materials, which depend on several parameters, such as the chemical composition, particle morphology, glass phase amount, particle size distribution, presence of inert materials, alkaline solution type, and process curing conditions. In addition, base materials with highly amorphous structures often contain glass beads, which have the capability to produce the alumina silicate at the low water levels [5,18]. A variety of alumina silicate materials have been exploited as raw materials in geopolymerization technologies [5], such as fly ash (F), blast furnace slag in granulated form, palm oil fuel ash, and rice husk ash. Fly ash, having pozzolanic properties, is a suitable source material, which can be acquired from thermal power plants via mechanical or electrostatic precipitation of the particles during the manufacturing process of geopolymers, because geopolymerization depends on the aluminosilicate chain. When higher contents of amorphous alumina and silica in the fly ash (especially class F) mix with an alkaline liquid, silicate species dissolve [19,20].

The type of activator and the concentration have substantial effects on the fly ash dissolution. Van Jaarsveld and Van Deventer [21] pointed out that leaching of Al^3+^ and Si^4+^ ions is commonly high for sodium hydroxide solutions in comparison to potassium hydroxide solutions. The typical content of the activator improves the mechanical properties of the geopolymer. Except with the typical concentration, substantial declines in the strength characteristics may occur as a result of the free OH^−^ in the alkali-activated matrix, which can be used to adjust the construction of the geopolymer material. Considering the mechanical strength, the effect of the concentration of the activator is greater than the effects of the curing temperature and the time [22,23,24,25,26]. According to Criado et al. [26], when free water glass was used together with NaOH as an alkaline activator, the polymerization process prompted a reaction product, in which more Si and better mechanical strength emerged. This statement was also supported by Fernandez-Jimenez and Palomo [27,28], who showed that compressive strength increases up to 40 to 90 MPa after one day of curing with NaOH and water glass rather than using only NaOH. Some researchers suggested that the use of a water glass–sodium hydroxide molar ratio of 2.5 enhanced the compressive strength of alkaline-activated fly ash [29,30]. However, Jumrat et al. [9] revealed that the workability of the geopolymer mortar was reduced by the Na_2_SiO_3_/NaOH and fly ash/alkaline solution ratios that were used, owing to the high water glass viscosity. The aggregate particle size pattern affects the flowability, as an increase in the ash/sand ratio enhances the workability of a geopolymer mortar [31]. Moreover, Wazien et al. [32] noted that the compressive strength of their geopolymer mortar increased when the binder/sand ratio increased from 0.25 to 0.5; however, the compressive strength showed a decreasing tendency when the binder/sand ratio changed from 0.5 to 4.0. 

Considering the studies in the literature on fly-ash-based geopolymer mortars, researchers have stated that the heat curing process is another significant parameter that affects the geopolymerization process. At ambient temperature, the geopolymer reaction takes place considerably slowly. Nevertheless, this reaction process speeds up with increasing temperature, and approximately 70% of the strength for a geopolymer is obtained within 3 to 4 h of heat curing [5,33,34,35,36,37]. It was proposed that the range of curing temperature determined between 30 and 90 °C ensures an effective geopolymer reaction [10,25]. Görhan and Kürklü [11] investigated the relationships between the alkali solution concentration, curing temperature, and curing time and the effects on the fly-ash-based geopolymers. To obtain the highest compressive strength for a geopolymer (22 MPa), they stated that the optimum thermal curing temperature and NaOH concentration were 85 °C and 6 M for samples cured up to 24 h and then kept at room temperature until the 7 day testing was performed. However, the study by Joseph and Mathew [38] indicated that the compressive, flexural, and splitting tensile strength values were 58, 4.95, and 4.51 MPa, respectively, for geopolymer cured at 100 °C for 24 h and tested at 28 days. Atiş et al. [35] obtained the highest compressive strength of 120 MPa for a fly ash geopolymer composite containing 14% sand-activated NaOH and cured at 115 °C for 24 h.

Regarding the development of green construction materials, the study of the production technology used for geopolymer mortars has continuously attracted researchers. Current discussions are focused on the impacts of the composition of the fly ash, the activator–binder and alkaline solution ratios, and the curing temperature on the geopolymerization reaction in the first 24 h of the process. For this purpose, different combinations of manufacturing parameters have been used to achieve optimal design parameters. Additionally, CEN reference sand was used in the design of the geopolymers in this study. Thus, experiments were carried out by reporting on the fresh performance, physical characteristics, and mechanical performance of the geopolymer mortars. For this purpose, two locally available waste materials, namely fly ash from the Çatalağzı Thermal Power Plant in Zonguldak and fly ash from İsken Sugözü Thermal Power Plant in Adana in Turkey, respectively, were employed as binders. Five different ratios of Na_2_SiO_3_/NaOH by weight, two activator/binder ratios, two different heat curing temperatures, and the fineness and composition of fly ash were considered as testing parameters. Simultaneously, to provide new insights into the literature, the differentiation of geopolymers owing to the chemical structures of the raw materials was investigated through scanning electron microscopy (SEM), X-ray diffraction (XRD), X-ray fluorescence spectrometry (XRF), and simultaneous thermogravimetric analysis (TGA).

## 2. Experimental and Numerical Analyses

### 2.1. Materials

**Figure 1:**
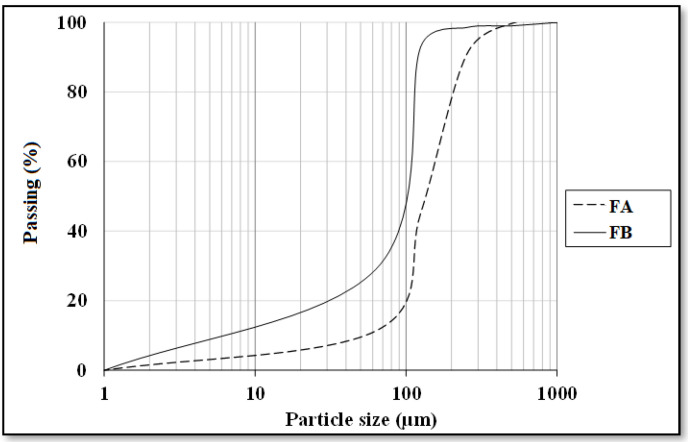
Particle size distribution of fly ashes.

Two different fly ash samples were provided from Çatalağzı Thermal Power Plant in Zonguldak (FA) and İsken Sugözü Thermal Power Plant in Adana (FB) in Turkey. Table 1 exhibits the physical and chemical properties of the fly ashes. Chemical analysis of the two fly ashes showed that both FA and FB contain more than 70% SiO_2_, Al_2_O_3_, and Fe_2_O_3_, concluding that both puzzolans could be categorized as class F type according to ASTM C618 [39]. Particle size analyses were also conducted by employing the method of laser diffraction, as shown in Figure 1. CEN reference sand was utilized as a fine aggregate. Moreover, the ratio of fly ash to sand was set to 1:1. The specific gravity and fineness modulus of the sand were 2.56 and 2.98, respectively. The alkaline activator was comprised of sodium silicate solution (Na_2_O = 14.7%, SiO_2_ = 29.4% and H_2_O = 55.9) and 12 M sodium hydroxide solution. NaOH solution was obtained by dissolving NaOH, which exist in the form of pellets.

### 2.2. Mix Proportion and Casting

In this research, 40 geopolymer mortars with different testing parameters were designed. Therefore, the effects of the liquid/solid ratio (fly ash + sand) (L/S), weight ratio of the alkaline activator, and curing temperature on the mechanical and physical characteristics of mortars were discussed. The details of the compound proportions utilized in the formation of the geopolymer mortar specimens are presented in Table 2. In the mortar mixture, the sand/fly ash ratio was kept constant. Two different L/S ratios were deployed in this study, which are listed as 0.4 and 0.8. In addition, Na_2_SiO_3_ with NaOH at ratios of 1.0, 1.5, 2.0, 2.5, and 3.0 by weight were used for geopolymer mortars. In this study, based on the material properties used in the production of geopolymer, the minimum curing temperature was obtained as 70 °C. Considering the minimum energy requirement in terms of sustainable development, the other curing temperature was determined as 100 °C to obtain the maximum strength characteristics. After the production stage for the geopolymer mortars listed in Table 2, the curing of the samples was completed at 70 °C and 100 °C in 24 h. Thus, 40 geopolymer mortars were produced with the different types of fly ashes and curing temperatures. The water-to-solids ratio for the produced geopolymer mortars was changed between 0.18 and 0.20, depending on the L/S ratio. The water content was given by the total water in the alkaline solution and additional water, while the solids were the sum of the fly ash, sand, and the solid part of the chemical activator. Additional water was used to obtain the desired workability, determined as 15 ± 2 cm.

The preparation process for the geopolymer mortar compounds was as follows. Initially, pellets of NaOH were dissolved in the water to reach the essential concentration. Afterwards, the sodium silicate and sodium hydroxide were combined with the desired amount of the alkaline solution in a mixer. Sequentially, fly ash and sand were added to the mixer and stirred for 5 min to achieve homogeneity. The freshly mixed mortar was fed into molds measuring 40 × 40 × 160 mm and vibrated in two layers until compaction was achieved. The specimens were covered with a heat-resistant thin plastic film to avoid evaporation. Finally, the geopolymer specimens were located in a laboratory-type furnace to cure thermally at different conditions for 24 h. The mortars presented in Table 2 were coded according to the testing parameters, such as the type of fly ash, the ratio of L/S, the ratio of Na_2_SiO_3_/NaOH, and the curing temperature. For example, consider the case which employs the L/S and Na_2_SiO_3_/NaOH ratios as 0.2 and 2.5 at a curing temperature of 70 °C. These geopolymer mortars were signified as 20FA-2.5-70, 20FB-2.5-70 for each type of fly ash. 

### 2.3. Testing Procedure

The workability of the fresh geopolymer mortars was assessed in accordance with ASTM C1437 [40] via a flow table test. After this process, the average diameter of the resulting fresh mortar spread was measured. Test batches were cast by adding water for each type of mortar to obtain the target slump flow diameter (15 ± 2 cm). Following the curing process completion, the samples were kept at room temperature for the mechanical and physical experiments until the testing age.

The morphological structure and microstructural properties of hardened geopolymers were investigated via SEM (Zeiss/Evo 40), EDX (EDAX/Ametek), XRD (Panalytical/Empyrean), thermogravimetric analysis (TGA-Linseis/PT1600), and differential thermal analysis (DTA-Perkin Elmer/DSC 400) at 28 days in the Niğde Ömer Halisdemir University Central Research Laboratory. To observe the effect of the design parameters on the microstructural analyses of the produced geopolymers, six specimens were examined. EDX data were obtained from the area where the SEM image was taken. XRD analysis was performed across a range of 10 to 70°. TGA analysis was practiced at a temperature scale of 25 °C to 1000 °C, with a scanning rate of 10 °C/min in a nitrogen atmosphere environment.

The bulk density, water absorption, and porosity values of the hardened mortars were determined according to ASTM C642 [41] by using prismatic specimens measuring 40 × 40 × 160 mm at 28 days. The average value from six measurements was calculated for every individual experiment and reported. 

The compressive and flexural strengths of geopolymer mortars were tested in agreement with ASTM C349 [42] and ASTM C348 [43] at 7, 28, and 90 days. Regarding the test ages, three prismatic samples measuring 40 × 40 × 160 mm were utilized. At first, the flexural strength test was carried out by calculating the average value of the outcomes received from three samples via a three-point flexural test. The compressive strengths of hardened mortars were measured using 6 test specimen pieces, which were broken in the flexural test. Finally, the compressive strength of the geopolymers was assessed using the average value of the outcomes received from 6 samples.

## 3. Test Results and Discussion

### 3.1. Workability of Geopolymers

**Figure 2:**
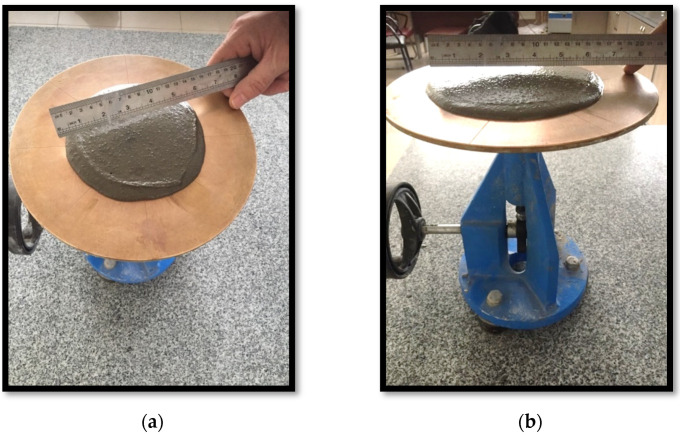
Flow diameter of (**a**) 20FA-1 and (**b**) 20FB-2 geopolymer mortars.

Generally, the flowability of fresh geopolymer mortars is controlled by the amount of water in the mix, without compromising the strength [44]. Less water results in higher dissolution rates for the aluminosilicate oxides. However, the amount of water should not fall below a certain value in order to retain the desired workability [31]. The dissolution rates were affected by the concentration of the Na_2_SiO_3_/NaOH solution. Ions contributed to the dissolution rates to balance the unstable geopolymer structure. Although lower amounts of OH^−^ enhance the dissolution rates, Na^+^ is still required, which comes from the alkaline activator solution. In the preparation of the sample, the workability for all samples was kept in the same range by taking the target slump flow diameter as 15 ± 2 cm, without adding superplasticizers (Figure 2). Thus, the workability of geopolymer mortars could be classified as moderate according to the flow diameter [23,45]. The flow diameter of the geopolymers produced from FB was greater than that of FA. As seen in Figure 2, 20FA-1 had a slump flow diameter of 15 cm, whereas 16.5 cm was observed for 20FB-2. The required water amount slightly increased through the increase of Na_2_SiO_3_/NaOH. This can be attributed to the high viscosity of the Na_2_SiO_3_ solution, which caused the rapid formation and higher viscosity of the fresh mortar [9]. Moreover, more water was supplemented to reach a fixed flow diameter for the geopolymer mortar of L/S = 0.2. Therefore, the flowability of the geopolymer mortar increased in accordance with the increment of the fly ash/alkaline solution ratio. Additionally, the workability and cohesiveness of the fresh geopolymer mortars resulted from the escalating ratio of SiO_2_/Na_2_O in the activator. For the second group (L/S = 0.4), the fixed flow diameter was obtained without adding water, in line with the literature [9,31].

### 3.2. Bulk Density, Water Absorption, and Porosity

The physical characteristics of hardened geopolymers are substantially different from those of the conventional mortars due to the distinct ingredients. Furthermore, the sand content of the geopolymer mortar substantially affects the bulk density, water absorption characteristics, and the porosity of the geopolymers [5,46]. Bulk densities were found to be in the ranges of 1355–1862 and 1574–1985 kg/m^3^ for the geopolymers produced from FA and FB, respectively. Based on the functional classification of ACI 318-11 [47], lightweight concretes can be classified into classes I, II, and III based on their compressive strength, density, and thermal conductivity values. Class I is structural lightweight concrete with a density range of 1440–1840 kg/m^3^, compressive strength >17 MPa, and thermal conductivity of 0.4–0.7 W/mK. Considering these criteria, almost all of the designed geopolymer mortars can be used for lightweight structural applications.

**Figure 3:**
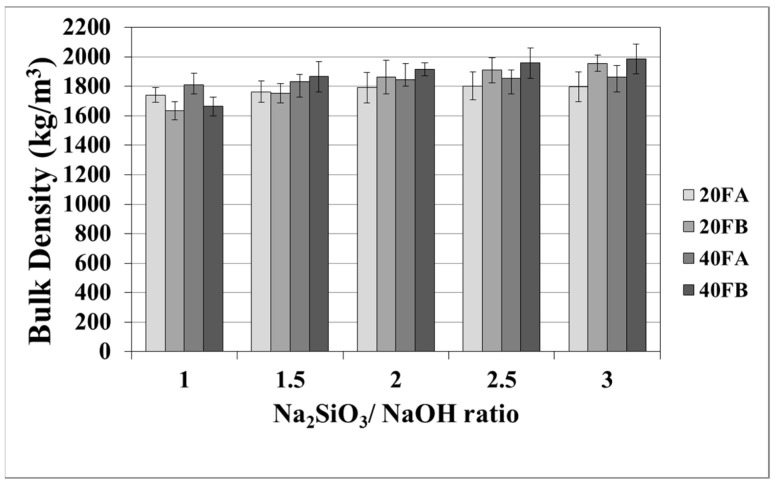
Bulk density values of geopolymer mortars cured at 70 °C.

**Figure 4:**
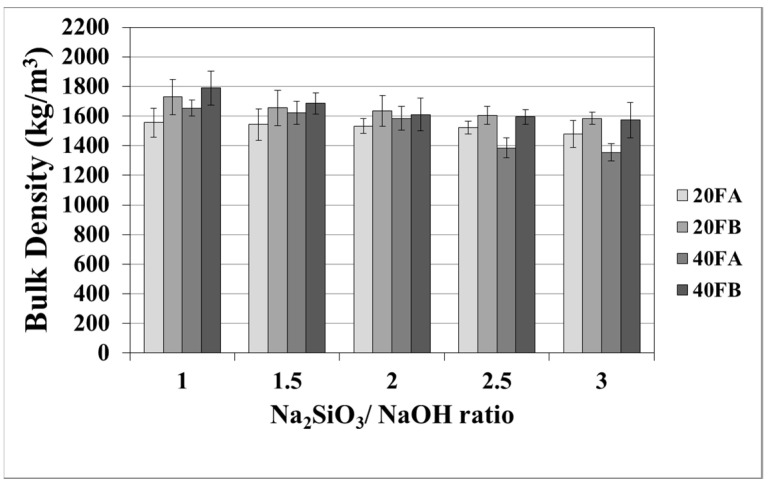
Bulk density values of geopolymer mortars cured at 100 °C.

The mortar made from FB, having finer particles, showed enhanced density in comparison to the mortar made from FA. The fineness or the particle size dispersion of F was one of the main parameters dictating the physical properties of geopolymer mortars [28,48]. As seen in Figure 3 in the samples cured at 70 °C, the bulk density of the hardened mortars was enhanced by the L/S and Na_2_SiO_3_/NaOH ratios. For this curing temperature, 40FB-3 achieved the highest density of 1985 kg/m^3^. This can be explained by the fact that the geopolymerization reaction is not fully completed at this curing temperature. It can be observed that for a given Na_2_SiO_3_/NaOH ratio, when the curing temperature was elevated, the bulk density of geopolymers declined for each type of F. Curing at high temperature (100 °C) resulted in a negating effect on the physical characteristics for expansion and tough geopolymer structures (Figure 4). This particular situation was also confirmed by Kong and Sanjayan [49], indicating that the change of homogeneity for the mortar due to fine aggregates caused differential thermal expansion within the aggregate and the paste. As a result, disintegration in the polymer network can occur. However, as demonstrated in Figure 4, increasing the L/S ratio at the curing temperature of 100 °C resulted in enhanced density for Na_2_SiO_3_/NaOH ratios of up to 2. An excessive amount of alkaline activator solution was another reason for the expansion of geopolymer mortars during the reaction. Therefore, the lowest density was obtained for mortar 40A-3 cured at 100 °C. Moreover, the water absorption rates of geopolymers reduced with increases in the bulk density.

**Figure 5:**
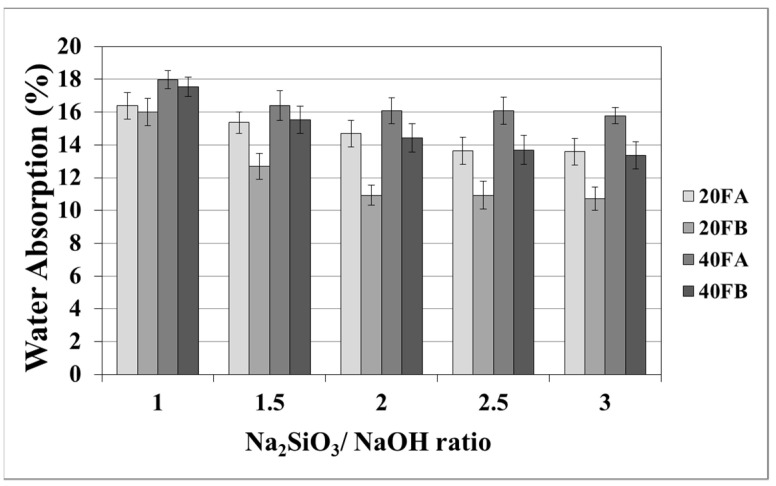
Water absorption values of geopolymer mortars cured at 70 °C.

**Figure 6:**
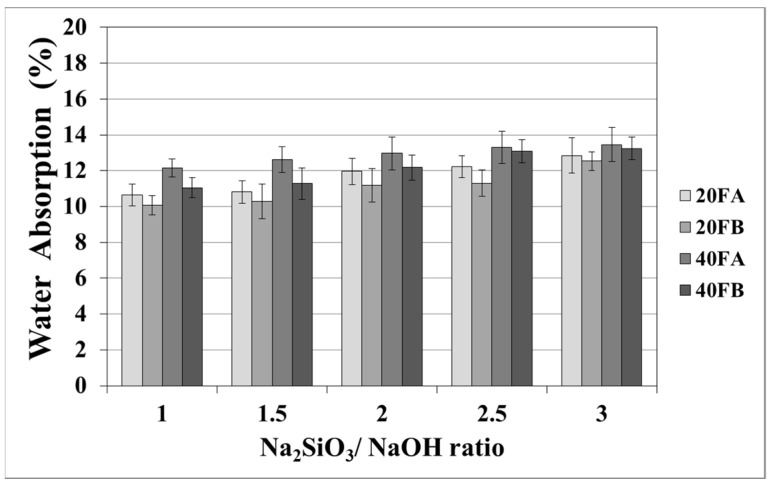
Water absorption values of geopolymer mortars cured at 100 °C.

As seen in Figure 5 and Figure 6, increasing the curing temperature resulted in a lower rate of water absorption. Additionally, water absorption rate reductions could be achieved by using a higher bulk density at a higher Na_2_SiO_3_/NaOH ratio at 70 °C curing temperature. In the sample, curing was completed at 100 °C for 24 h. The pores were filled with reaction products formed during the geopolymer reaction process. For instance, while the lowest water absorption rate was observed as 10.07% for 20FB-1-100, 40FA-1-70 had the highest water absorption (17.98%) capacity. 

**Figure 7:**
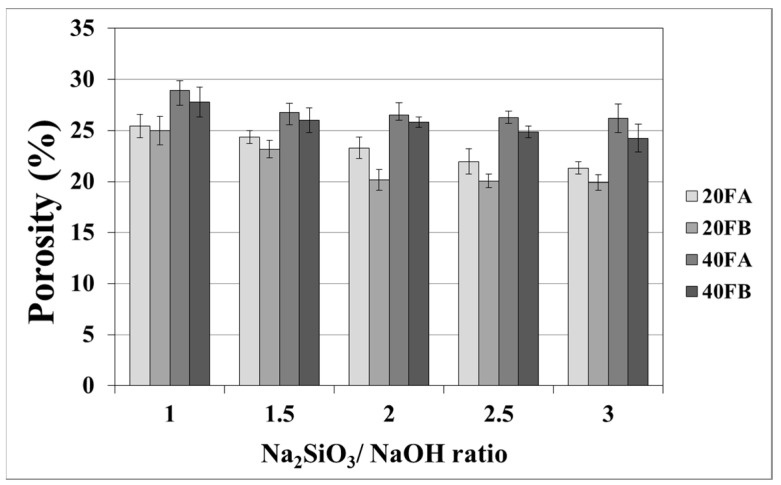
Porosity of geopolymer mortars cured at 70 °C.

**Figure 8:**
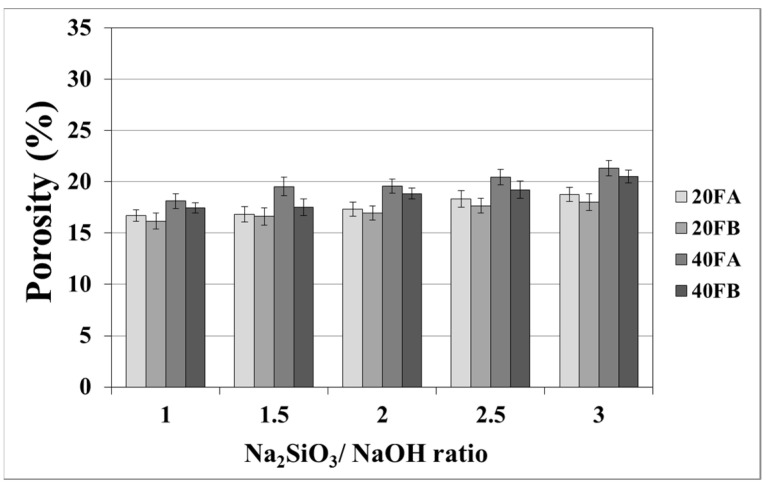
Porosity of geopolymer mortars cured at 100 °C.

As seen in Figure 7 and Figure 8, the porosity values of geopolymers presented a similar trend in terms of water absorption values. Since porosity decreased with the decreasing pore size, the water absorbed by mortars also decreased. Hardened geopolymer mortars produced with an L/S ratio of 0.4 experienced a higher amount of water absorption and apparent porosity in comparison to the ones manufactured with a lower L/S ratio at a low curing temperature. For instance, 20A-3 showed a capacity for water absorption of 13.58%, while the water absorption capacity was found to be 15.78% for 40A-3 at a curing temperature of 70 °C. As seen explicitly in Figure 7 and Figure 8, the porosity of the samples cured at 70 and 100 °C ranged from 19.93% to 28.96% and from 16.16% to 21.33%, respectively. Similarly, Görhan and Kürklü [11] obtained comparable porosity values of 25–30% for fly ash geopolymers. Moreover, the bulk density of geopolymer mortars designed with an L/S ratio of 0.4 was higher than that of geopolymer mortars designed with an L/S ratio of 0.2. A similar pattern was also seen for the apparent porosity at the curing temperature of 70 °C. Similar situations were also noticed by other authors [11], which can be explained by the fact that the total porosity of the samples at the L/S ratio of 0.4 is higher than that of samples with a lower L/S ratios. In line with the literature, this study showed that the geopolymer mortar, which is produced with a higher alkali content (% Na_2_O) and cured at a higher temperature, had a lower water absorption rate and apparent porosity values [33,34,35,36]. Similarly, regardless of the L/S ratio and based on the bulk density values of hardened mortars, the water absorption and the apparent porosity increased with escalating Na_2_SiO_3_/NaOH ratios for the samples cured at 100 °C. This may be associated with the fact that the thermal expansion occurred between the aggregate and paste due to the high curing temperature. As a consequence of the physical tests, the most decreased water absorption rates and porosity values were observed in the geopolymers with an L/S ratio of 0.2, which were cured for 100 °C.

### 3.3. Compressive and Flexural Strengths

**Figure 9:**
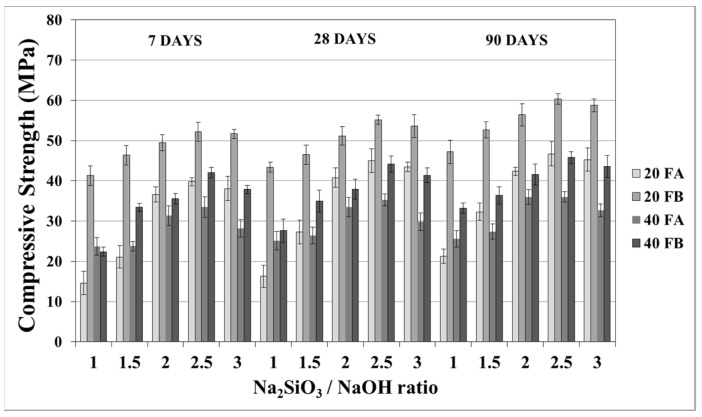
Compressive strength of geopolymer mortars made from FA and FB produced at 70 °C.

**Figure 10:**
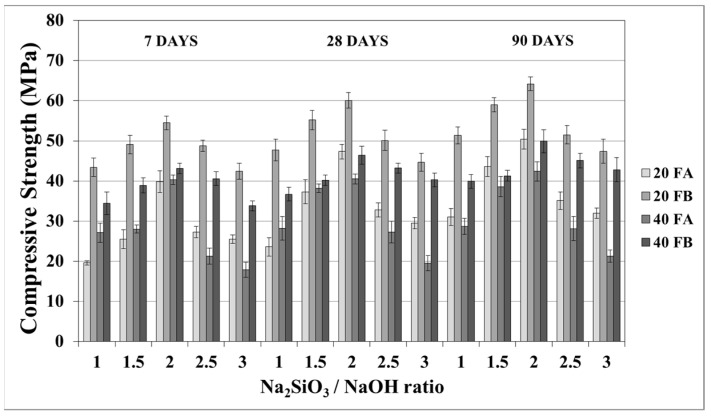
Compressive strength of geopolymer mortars made from FA and FB produced at 100 °C.

The compressive strength is the most common property used to describe the design properties of fly ash-based geopolymers. Figure 9 and Figure 10 indicate the compressive strength values of the geopolymer mortars with different design parameters. As seen from these figures, the variations in compressive strength increased slightly after 7 days; thereafter, the strength gained between 7 and 90 days was insignificant. Van Jaarsveld et al. [21] reported that the strength of a geopolymer cured for 4 h can reach 70% of its ultimate strength. It was observed that approximately 68–99% of the ultimate strength was obtained at the age of 7 days in this study. Therefore, the range of compressive strength was in good agreement with the literature, which showed that the compressive strength of geopolymers does not significantly change with age [5,27,45]. As seen in Figure 9 and Figure 10, the compressive strength of the hardened mortars changed between 16.3 and 47.3 and between 27.6 and 60.1 MPa for FA and FB, respectively, at 28 days. Considering these values, as mentioned for the bulk density, the geopolymer mortars designed in this study can be used for structural lightweight applications based on the ACI 318-11 [47] criteria. The low soluble amounts of Al and Si in the FA sample was the reason for the lower compressive strength compared to that of the FB sample. The fineness of the pozzolans could also affect the compressive strength. Accordingly, 20FB-2.5-70 and 20FB-2-100 mortars had the highest compressive strength in all cases. It can be attributed that geopolymer mortars made from FB have the highest content of fine particles. This conclusion was supported by Komljenovi’ et al. [48], who indicated that smaller FB particle sizes (smaller than 43 µm) were more active in the reaction. The L/S ratio is effective in causing variations of the compressive strength, as seen in Figure 9 and Figure 10. For example, it can be observed in Figure 9 that when the L/S ratio increased from 0.2 to 0.4, a slight increment in the compressive strength could be obtained up to the limited value of the alkaline solution for the geopolymer produced with FA, but not for FB. Thus, it can be concluded that the alkaline activator ratio is the most dominant parameter affecting the strength characteristics during geopolymerization. Additionally, as observed in Figure 9 and Figure 10, a higher curing temperature resulted in increased compressive strength for mortars with different Na_2_SiO_3_/NaOH ratios, whereas this trend was not observed for the other specimens with Na_2_SiO_3_/NaOH ratios greater than 2. 

The increased compressive strength at higher curing temperatures can be explained by the improvement of kinetic energy resulted in the geopolymer mortars to have stronger Al-Si-O networks [50]. Additionally, at a constant curing temperature, an increase of the Na_2_SiO_3_/NaOH ratio caused an increase in the compressive strength up to a certain value (Na_2_SiO_3_/NaOH = 2). It is known that a higher amount of soluble silica in the geopolymerization delays the dissolution of F due to improvement of the saturation of ionic silica species, while the promotion of the precipitation of larger molecular species results in a stronger gel [51]. The reaction kinetics and rate of crystallization are directly influenced by the presence of soluble silica. This encourages a Si-rich gel formation, which is responsible for the development of material strength [52]. Additionally, the formation potential of the aluminosilicate gel, which contributes to the mechanical strength of geopolymer mortar, is caused by the presence of highly reactive silica in the F [53]. For these reasons, 20FB-2-100 had the highest compressive strength in this study. After a certain Na_2_SiO_3_/NaOH ratio, the reduction of compressive strength was attributed to an increase in the coagulation of silica [35,36]. Moreover, it may have been due to the excess of OH^−^ in the mixture. 

Atmospheric carbonation can produce sodium carbonate from the excess sodium concentration, which may affect the polymerization [52,53,54]. Thus, as observed in Figure 9 and Figure 10, significantly lower strength was noticed after the Na_2_SiO_3_/NaOH ratios of 2.5 and 2 for the curing temperatures of 70 °C and 100 °C, respectively. This conclusion was supported by Yang et al., 2012 [55], who indicated that using excessive activator solutions resulted in superfluous water or a less alkali reaction, which in turn weakened the bonding and decreased the mechanical properties of geopolymers. Similarly, Khale and Chaudhar [6] noted that there was an optimum concentration limit for each activator type. Exceeding this limit results in the reverse effects for the strength characteristics. Moreover, as observed for mixtures 20FB-2.5, 20FB-3, 40FB-2, and 40FB-3, elevated curing temperatures conspicuously decreased the compressive strength of the specimens with Na_2_SiO_3_/NaOH ratios greater than 2. It seems that the curing time of 24 h was not long enough for the strength development for these samples. However, Atis et al. [35] stated that the water in the mixture could be immediately vaporized by the increasing temperature without allowing for the dissolution of silica in F.

**Figure 11:**
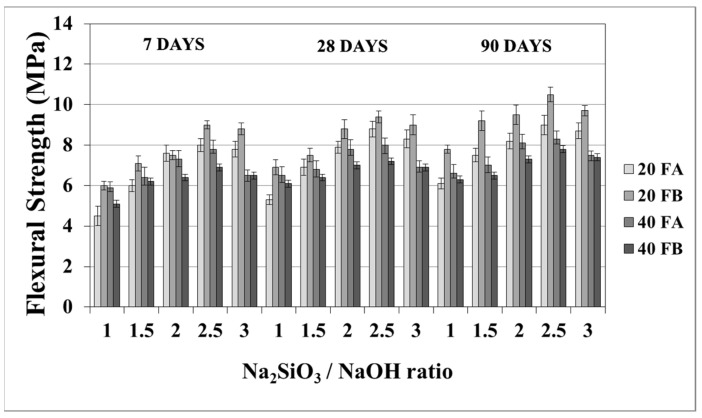
Flexural strength values of geopolymer mortars made from FA and FB and produced at 70 °C.

**Figure 12:**
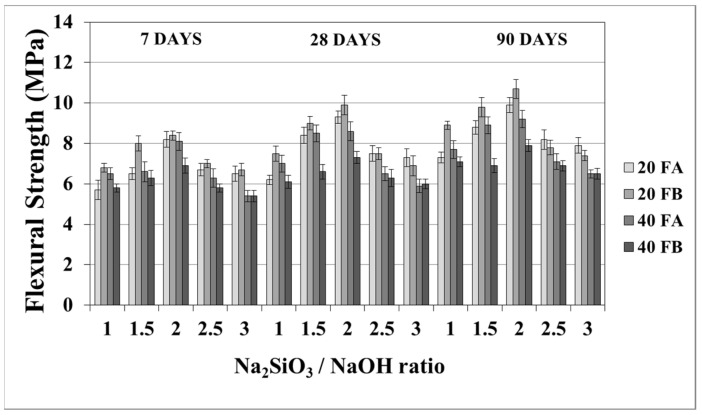
Flexural strength values of geopolymer mortars made from FA and FB and produced at 100 °C.

The flexural strength values at 7, 28, and 90 days are indicated in Figure 11 and Figure 12. The overall flexural strength of the geopolymers slightly increased with the testing duration, as can be observed from the compressive strength results. This could be a consequence of the denser structure of the hardened mortar, which showed improved crystallinity from day 7 to day 90. As seen in Figure 11 and Figure 12, the flexural strengths changed between 5.3 and 9.9 MPa. Furthermore, the hardened mortars designed with the L/S ratio of 0.4 exhibited lower flexural strength values in comparison to the L/S ratio of 0.2. This can be explained by the higher water/cementitious materials ratio, which can cause a reduction in the strength values of the Portland cement paste [56]. Thus, it can be observed from Figure 11 that the 40FA-1 mortar cured at 70 °C for 24 h had the lowest flexural strength. Similar to the compressive strength test results, the flexural strength increased up to a certain value along with the Na_2_SiO_3_/NaOH ratio. Alkalis, which are responsible for the dissolution of solid F particles, regulated the geopolymer reaction. However, the degree of polymerization of the dissolved gel was affected by the soluble silicates. Nevertheless, when excessive silicates were used, the strength characteristics were deteriorated by the delayed geopolymer reactions, which are the result of the coagulation of silica [11,35,49]. Therefore, the highest flexural strength values were found to be 9.3 and 9.9 MPa for 20FA-2-100 and 20FB-2-100, respectively, at 28 days. Despite the increased curing temperature, it was concluded that reductions of flexural strength occurred at Na_2_SiO_3_/NaOH ratios higher than 2.

### 3.4. SEM, EDX, XRD, and TGA-DTA Investigations

The reaction kinetics of fly-ash-based geopolymers can be governed by different factors, such as the source of the fly ash, the non-uniform reaction of the fly ash with different alkali amounts, high amounts of impurities in the reaction system, the range of temperatures used for the reaction, and the properties of the products derived from the unreacted fly ash and alkalis in the system. Here, 40 geopolymers were considered in total and the effects of these parameters were observed. When the fly ash was reacted with an alkali, nucleation and chemical reactions on the surfaces of the particles occurred at first. The compositions of FA and FB are the major influencing factors in this step. First of all, in this study, the geopolymer mortars designed with FB had better strength characteristics than those made from FA due to the higher SiO_2_/Al_2_O_3_ ratio. Then, the second mechanism was followed by the phase boundary interaction–diffusion of reactants through a porous layer of the reaction products. This step could be improved by applying a higher curing temperature. However, increments of the L/S ratio for 0.2 to 0.4 did not significantly change the results (the 40FA and 40FB groups). Finally, a dense layer formed by the reaction products. For the last step, the ratio of Na_2_SiO_3_ to NaOH in the activator solution was crucial for the dissolution of Si^4+^ and Al^3+^ to form the nM_2_O.Al_2_O_3_–xSiO_2_.yH_2_O matrix [57]. Increasing this ratio up to 2 for the geopolymer mortar enhanced the optimum alkalinity. Thus, the reaction kinetics could be modified via the presence of soluble silica in these mixtures, thus improving the compressive strength [58]. These observations of the better strength characteristics were proven by the denser structure, high Si/Al ratio, higher amorphous phases, higher N-A-S-H content, as assessed using SEM, EDX, XRD, and TGA-DTA tests, respectively, for the geopolymer mortars.

Considering the strength values, 20FA-1-70, 40FA-1-70, 20FA-2-70, 20FB-1-100, 40FB-1-100, and 20FB-2-100 were selected in order to observe the effectiveness of the design parameters of the hardened geopolymers using SEM, EDX, XRD, and TGA-DTA tests during the geopolymerization process. 

**Figure 13:**
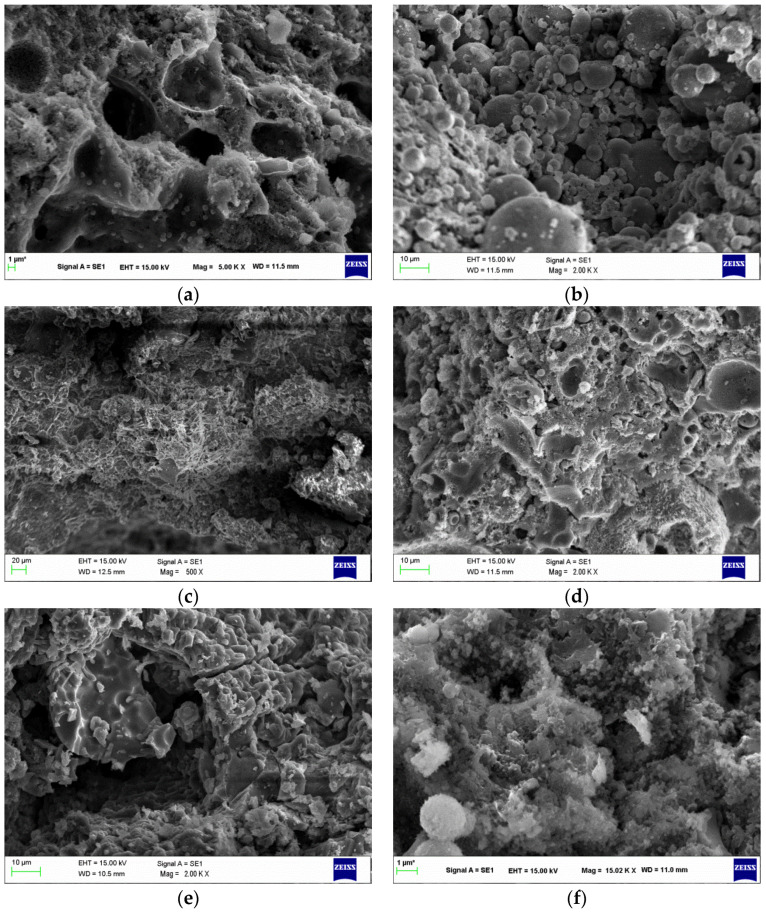
SEM images at 28 days for (**a**) 20FA-1-70, (**b**) 40FA-1-70, (**c**) 20FA-2-70, (**d**) 20FB-1-100, (**e**) 40FB-1-100, and (**f**) 20FB-2-100. (Specimen types exhibit different characteristics due to the production parameters; thus, different SEM magnitudes were utilized to capture images).

Figure 13 illustrates the SEM analysis of F-based geopolymers. The composition of F, L/S ratio, Na_2_SiO_3_/NaOH ratio, and curing temperature had certain effects on the microstructural development and morphology of the reaction products. The saturation degree rate of the ionic species and the strength of the mortar were affected by the amount of alkaline solution in the system [52]. It can be observed that the SEM image of the 20FB-2-100 sample is remarkably compacted and dense, which shows that the increase in the Na_2_SiO_3_/NaOH ratio up to a certain limit increases the compactness. Additionally, Figure 13 shows that the geopolymers synthesized with FB exhibited the most homogeneous and least porous microstructures, which was attributed to the high dissolution of the fly ash particles and high SiO_2_/AL_2_O_3_ ratio in the alkaline activator solution. Moreover, similar to previous studies, the SEM results for mortars showed unreacted or partially reacted spherical F residues and a continuous mass of aluminosilicate [15]. The unreacted reactants of FA can be seen in 20FA-1-70 and 40FA-1-70, respectively. 20FA-1-70 also had showed extensive formation of the pores. As can be seen from Figure 13, the microspheres of 20FA-1-70 and 40FA-1-70 were confined within a crust of reaction products. The bond of the crust to the fly ash particles did not seem to be very strong due to the low Na_2_SiO_3_/NaOH ratio and curing temperature. However, the 20FA-2-70 mixture with a higher Na_2_SiO_3_/NaOH ratio did not show spherical shapes for the FA. As a result of the increase in the Na_2_SiO_3_/NaOH ratio, all F particles in the mixture were involved in the reaction. This was the main reason for the increase in strength. Significantly denser microstructures with lower porosity were obtained for 20FB-1-100, 40FB-1-100, and 20FB-2-100 as compared to 20FA-1-70,40FA-1-70, and 20FA-2-70 due to the highly reactive silica in the F, the curing temperature, and the higher filling ability of the former samples.

**Figure 14:**
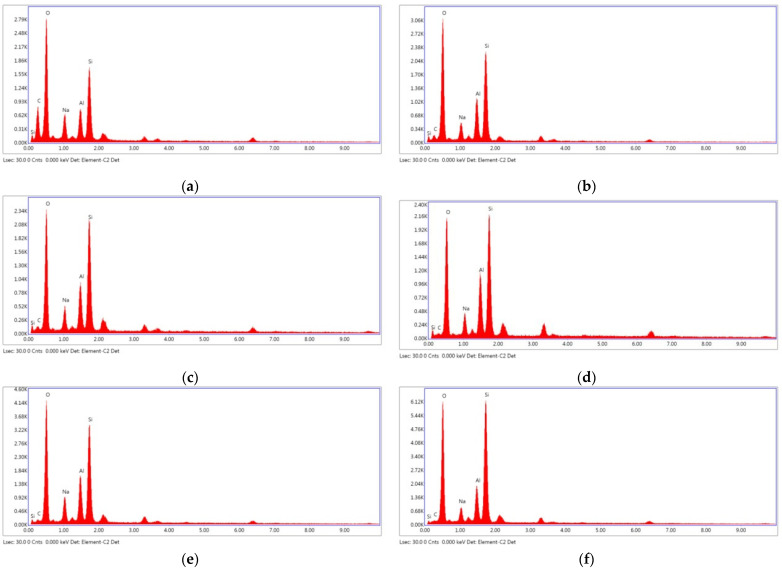
EDX analysis at 28 days for (**a**) 20FA-1-70, (**b**) 40FA-1-70, (**c**) 20FA-2-70, (**d**) 20FB-1-100, (**e**) 40FB-1-100, and (**f**) 20FB-2-100.

According to the EDX spectrum, the major components of the fly ash geopolymer paste were Si and Al, with small amounts of Na and K [15,59]. The Si/Al ratios as observed from the EDX analysis results were within the suggested range of 1–3 [8]. As can be observed in Figure 14a,c,d,f, the increased Na_2_SiO_3_/NaOH ratio increased the silica content and Si/Al ratio of the mixture. Therefore, 20FA-2-70 and 20FB-2-100 mixtures had higher compressive strength than 20FA-1-70 and 20FB-1-100 mixtures. In addition, as can be seen in Figure 14, FB, which had more reactive silica compared to FA and a higher temperature, provided gels that were richer in Si, thus resulting in a greater degree of geopolymerization. For example, the 20FB-1-100 sample had much more Si than the 20FA-1-70 sample. The aluminosilicate compound of lower strength was produced by reducing the Si/Al ratio, which was accompanied by a microstructure with an increased crystalline phase [8]. This was the reason for the high strength difference between the two mixtures. The geopolymer paste with the higher SiO_2_/Al_2_O_3_ ratio, as seen in the SEM photos, showed the presence of a continuous and dense mass of strong geopolymer.

**Figure 15:**
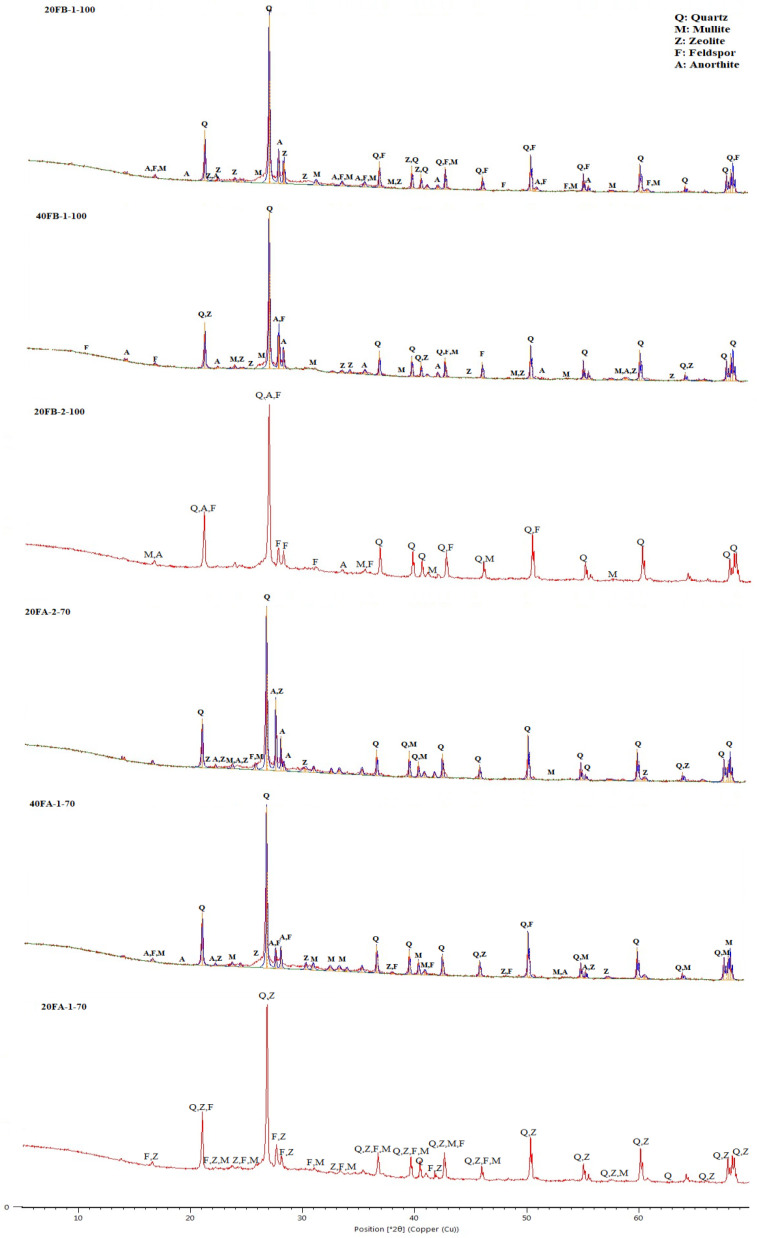
XRD analysis of hardened mortars (20FA-1-70, 40FA-1-70, 20FA-2-70, 20FB-1-100, 40FB-1-100, and 20FB-2-100).

The XRD trends for 20FB-1-100, 40FB-1-100, and 20FB-2-100 geopolymers were similar to those of 20FA-1-70, 40FA-1-70, and 20FA-2-70. As shown in Figure 15, the results of the XRD analysis of the alkali-activated FA samples indicated that a large quantity of quartz and a small amounts of mullite and feldspar (albeit, anorthite) were associated with the crystalline phases. The amorphous phases were defined in a wide and diffusive reflection in the interval range from 25° to 35° 2*θ*. Zeolite phases were noticed, especially in the specimens of 20FA-1-70 and 40FA-1-70 when a decreasing Na_2_SiO_3_/NaOH ratio and curing temperature were deployed. However, this type of zeolite was not found when the same FB was activated with the Na_2_SiO_3_/NaOH ratio of 2 and cured under the same conditions (20FB-2-100) [48]. The synthesis conditions of the geopolymer products, which can result in crystal formation, are important for assessing the desired impacts on the chemical and physical characteristics. Whilst the possibility of the formation of the zeolites was reduced by an increment of the SiO_2_/Na_2_O ratio in the solution, the amorphous phases became the only reaction products [60]. The geopolymerization of FA necessitates the utilization of rather concentrated alkali solutions with a rather low L/S ratio, whereas the nuclei of the zeolites are comprised of the reaction products. However, the enlargement of the zeolite crystals was very minor; hence, the aluminosilicate gel was initially stable. The absence of crystalline products was closely related to the higher compressive strength [48]. As can be observed in Figure 15, the minimization of the zeolite led to higher compressive strength for 20FB-2-100. Compared to 20FB-2-100, sharp peaks of mullite and zeolite were observed in 20FA-1-70, indicating that SiO_2_ and Al_2_O_3_ in F were not fully consumed in the geopolymerization process [61]. The feldspar peak (albeit, anorthite) for 20FB-2-100 may have contributed to the high strength of the geopolymer mortar by forming a crystalline phase of the N-A-S-H (aluminosilicate gel) system [48]. Moreover, as seen for 20FB-1-100, 40FB-1-100, and 20FB-2-100, the entity of the crystalline peaks promotes the development of the strength of the geopolymer mortar [62]. The quantities of quartz and mullite in the geopolymer samples showed remaining amounts that were fully reacted during the repolymerization process for the testing parameters.

**Figure 16:**
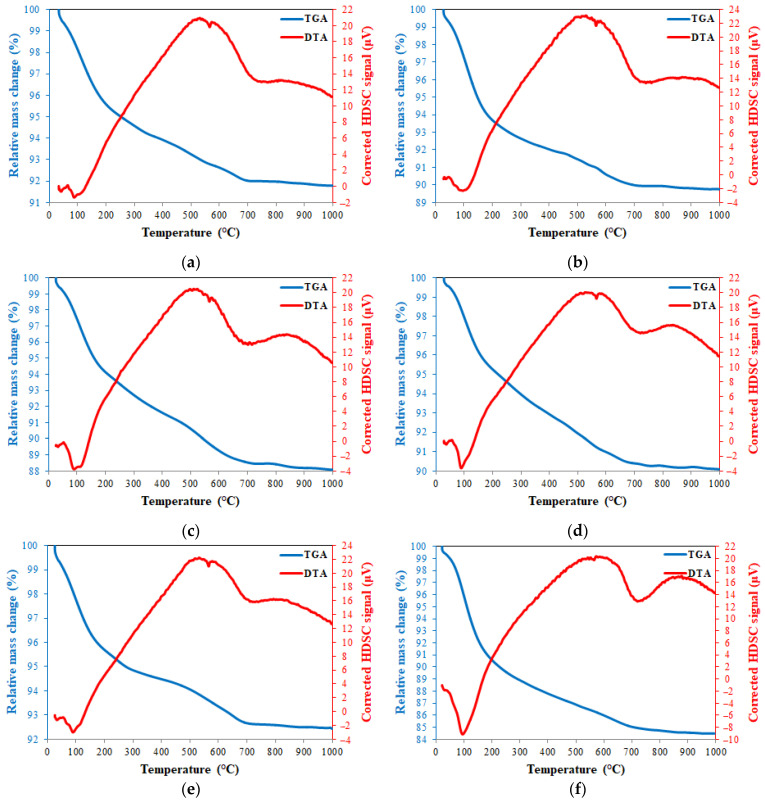
TGA and DTG thermograms at 28 days for (**a**) 20FA-1-70, (**b**) 40FA-1-70, (**c**) 20FA-2-70, (**d**) 20FB-1-100, (**e**) 40FB-1-100, and (**f**) 20FB-2-100.

TGA and DTA thermograms of 20FA-1-70, 40FA-1-70, 20FA-2-70, 20FB-1-100, 40FB-1-100, and 20FB-2-100 are presented in Figure 16. The loss of the water due to evaporation of free water, which is absorbed and trapped, was demonstrated with a sharp reduction in mass under 250 °C [49,63,64]. The continuous mass loss from 200 °C to 800 °C resulted in the evaporation of chemically bonded water, as well as the condensation of the hydroxyl group. These findings were also supported by a study in the literature [65]. However, an increased rate of mass loss with the increase in temperature from 800 °C to 1000 °C was detected. In agreement with Kong and Sanjayan [49] and Abdulkareem et al. [63], the average of the mass temperature when further heated to 800 °C approximately changed between 85–92% for the tested geopolymer mortars, as reported in Figure 16. The stabilized region from 800 °C to 1000 °C showed no crystallization or phase transitions caused by the considerable heat effect [66].

The peaks of the endothermic characteristics were fixed at between 30 and 165 °C, which was related to the phase formation of sodium aluminium silicate. Observing the similarity of the results to the previous studies, the endothermic peaks of the samples located at 723–728 °C were caused by the formation of carbonates and sodium carbonates. Silicon and aluminum phases occurred between 600 and 800 °C [67]. Thus, the high heating process for secondary silicate phases may be occurred due to the endothermic peak at around 700 °C, resulting in the intensification of the geopolymer paste, which was considered the cause of the strength gain of the geopolymer mortar at elevated temperature [68]. According to this information, for the six analyzed samples shown in Figure 16, the peak around 100 °C represents the N-A-S-H system and the peak around 700 °C shows NaCO_3_. As can be seen in Figure 16, the N-A-S-H content of 20FB-2-100 was higher than in the other mixtures. The N-A-S-H content of the 20FA-1-70 (Figure 16a) mixture was lower than that of the 20FA-2-70 (Figure 16c) mixture. Similarly, the N-A-S-H content of the 20FB-1-100 (Figure 16d) mixture was lower than the 20FB-2-100 (Figure 16f) mixture. This situation may explain why the lower Na_2_SiO_3_/NaOH ratio reduces the N-A-S-H ratio of the geopolymer. In addition, it can be said that the utilization of a geopolymer mixture of fly ash with higher SiO_2_ content (FB) with the increased temperature (100 °C) improved the amount of N-A-S-H in the geopolymer. For example, the N-A-S-H contents of the 20FB-1-100 (Figure 16d) and 20FB-2-100 (e) mixtures were higher than the 20FA-1-70 (Figure 16a) and 20FA-2-70 (c) mixtures, respectively. Therefore, the N-A-S-H peak in the DTA diagram changed in parallel with the compressive strength. Thus, the results obtained from this analysis are supported by the mechanical test results. 

When the strength values of the geopolymers based on the results of compressive and flexural strength results were evaluated, it was concluded that the 20FB-2 sample cured at 100 °C for 24 h had higher values than the other geopolymers. This suggests that a decrease in the Si/Al ratio could lead to lower strength characteristics for 20FA-1-70 with respect to EDX analysis. Increase in the Na_2_SiO_3_/NaOH weight ratio up to a certain limit resulted in higher Si contents with respect to the curing temperature. The TGA-DTA analysis results showed that DTA peaks for N-A-S-H mixtures with higher compressive strength had higher intensity. Moreover, the physical and mechanical properties were supported by SEM, EDX, XRD, and TGA-DTA tests.

## 4. Conclusions

Based on the findings of this study, the following conclusions can be drawn:

- This study showed that geopolymer mortars can be manufactured through activation of locally available class F fly ashes (FA and FB) with different alkaline solution types and contents. The acceptable workability was classified as moderate based on the slump flow diameter (15 ± 2 cm), which was obtained for all geopolymer mixtures without adding superplasticizers.
- The chemical compositions of source materials for the geopolymers should be rich in silicon and aluminum oxides. These oxides in class F fly ash react with liquid alkaline to produce geopolymer mortars that bond the fine aggregates or unreacted particles. Considering the test results, the geopolymer mortars designed with FB had gave results than those of FA due to the ratio of silicon oxide (SiO_2_) to aluminum oxide (Al_2_O_3_) by mass of the raw material, which should preferably be in the range of 2.0 to 3.5 to produce geopolymers with better strength characteristics.
- The highest bulk density was 1985 kg/m^3^ for 40B-3-70, whereas 40A-3-100 gave the lowest value (1355 kg/m^3^). Considering FA and FB, the fineness or particle size distribution of F was one of the main parameters in terms of the physical characteristics of the geopolymer mortars, as well as the Na_2_SiO_3_/NaOH and L/S ratios. The bulk density was increased by the ratios of L/S and Na_2_SiO_3_/NaOH at low curing temperatures due to the geopolymerization reaction not being fully completed at such curing temperatures for the hardened mortars. However, curing at high temperature (100 °C) resulted in a reduction in the bulk density due to the expansion and tough structure of the geopolymer samples.
- The water absorption and porosity values changed between 10.07% and 17.98% and between 16.71% and 27.77%, respectively. Hardened geopolymer mortars produced with an L/S ratio of 0.4 resulted in higher values of water absorption and apparent porosity when compared to the ones produced with a lower L/S ratio based on the increases in the alkaline solution and water. However, regardless of the L/S ratio, the apparent porosity and the water absorption increased with the increasing ratio of Na_2_SiO_3_/NaOH for the samples cured at 100 °C due to the geopolymerization.
- The highest compressive and flexural strength values were found to be 60.1 MPa and 9.9 MPa for the mixture with the L/S ratio of 0.2 cured at 100 °C for 28 days. It can be concluded that using excessive activator solutions caused less alkali reaction, which weakened the bonding in the geopolymer matrix. On the other hand, the decrease in mechanical properties of geopolymers after using the optimal Na_2_SiO_3_/NaOH ratio was attributed to an increase in the coagulation of the silica.
- The heat curing process substantially affected the chemical reaction that occurs in the geopolymer mortar based on the type of fly ash. Higher curing temperatures resulted in better compressive strength for both fly ashes due to the improvement of kinetic energy, as well as the reaction degree, which caused the geopolymer mortars to have a stronger Al-Si-O network.
- Considering all of the test results, in order to obtain the best strength characteristics, the curing temperature, L/S ratio, and Na_2_SiO_3_/NaOH should be 100 °C, 0.2, and 2.5, respectively, for FA, whereas a curing temperature of 100 °C, L/S ratio of 0.2, and Na_2_SiO_3_/NaOH ratio of 2 were optimal for FB.
- SEM analysis indicated that the compact composite matrix was enhanced for 20FB-2-100, which had the highest compressive strength among the tested samples. The EDX analysis showed that FB with more soluble silica, increased temperature, and an increased Na_2_SiO_3_/NaOH ratio improved the mechanical performance by providing a better geopolymerization reaction. According to the XRD analysis, SiO_2_ and Al_2_O_3_ in F for the 20FA-1-70 mixture did not fully take part in the geopolymerization, which resulted in lower compressive strength. In addition, DTA peaks observed in the range of 30–165 °C supported the mechanical tests by showing that the N-A-S-H peak increased with the increased compressive strength.
- The production of fly-ash-based geopolymers not only provides an opportunity to use F as a sustainable green material via alkaline activation but also avoids the disposal problems associated with F. Additionally, this method contributes to the environment by decreasing CO_2_ emissions into the atmosphere, since it is a cement-free technology. Thus, geopolymers manufactured in this study with the desired strength characteristics can be used as brick building blocks, tiles, and prefabricated materials, as well as in infrastructure works.

## Figures and Tables

**Table 1:** Physical and chemical properties of fly ashes.

Analysis Report (%)	FA	FB
CaO	2.00	1.53
SiO_2_	54.08	62.28
Al_2_O_3_	26.08	21.46
Fe_2_O_3_	6.68	7.01
MgO	2.67	2.37
SO_3_	0.73	0.07
K_2_O	4.53	3.81
Na_2_O	0.79	0.26
Others	2.44	1.21
Loss on ignition (LOI)	1.52	1.78
Specific gravity	2.04	2.25
BET (m^2^/g)	1.11	2.26

**Table 2:** Mix proportions for geopolymer mortars.

Fly Ash (g)	Sand (g)	Liquid/Solid Ratio	Na_2_SiO_3_/NaOH Ratio	NaOH (g)	Na_2_SiO_3_ (g)	Total Water/Solid Ratio
600	600	20%	1.0	120	120	0.18
			1.5	95	144	
			2.0	80	160	
			2.5	68	172	
			3.0	60	180	
		40%	1.0	240	240	0.20
			1.5	192	288	
			2.0	160	320	
			2.5	136	344	
			3.0	120	360	

## Data Availability

No new data were created or analyzed in this study. Data sharing is not applicable to this article.
